# Congenital Pulmonary Airway Malformation of the Left Upper Lobe Associated With Dextrocardia in a Neonate: A Case Report

**DOI:** 10.7759/cureus.108103

**Published:** 2026-05-01

**Authors:** Khadija Mesbah, Kaoutar Ettoini, Kawtar Khabbach, Yousra El Boussaadni, Abdallah Oulmaati

**Affiliations:** 1 Pediatrics, Centre Hospitalier Universitaire Mohammed VI de Tanger, Tangier, MAR

**Keywords:** congenital pulmonary airway malformation, cystic lung lesion, mediastinal shift, neonatal respiratory distress, pseudo dextrocardia

## Abstract

Congenital pulmonary airway malformation (CPAM) is a rare developmental lung anomaly that may present in the neonatal period with respiratory distress, and its association with dextrocardia is exceptional, potentially leading to diagnostic challenges.

We report the case of a 26-day-old male neonate, born at term, admitted for cyanosis and respiratory distress. Clinical examination revealed tachypnea (68 breaths per minute), oxygen desaturation (SpO₂ 65% in room air), and signs of respiratory distress, with cardiac auscultation suggesting right-sided heart sounds. Imaging studies demonstrated a cystic lesion of the left upper lobe consistent with congenital pulmonary airway malformation, associated with cardiac dextroposition due to mediastinal shift, rather than true dextrocardia. The patient received supportive care and broad-spectrum antibiotic therapy; however, the clinical course was marked by progressive respiratory deterioration complicated by severe pulmonary infection, ultimately resulting in death before surgical intervention could be undertaken. CPAM results from abnormal airway development, and it is associated with cardiac dextroposition due to mediastinal shift, rather than true dextrocardia. This case highlights the importance of considering CPAM in neonates presenting with respiratory distress and emphasizes the crucial role of imaging, particularly computed tomography, in establishing the diagnosis and guiding management.

## Introduction

Congenital pulmonary airway malformation (CPAM), formerly known as congenital cystic adenomatoid malformation, is a rare developmental anomaly of the lower respiratory tract resulting from abnormal airway branching during fetal lung development [1,2]. It accounts for approximately 20-25% of congenital pulmonary malformations [3]. Histologically, CPAM is characterized by abnormal proliferation of terminal bronchioles forming cystic or adenomatous structures. The Stocker classification remains the most widely used system to categorize these lesions, with type I being the most common form and typically associated with large cysts [4].

The clinical presentation of CPAM is highly variable, ranging from asymptomatic cases detected prenatally to severe neonatal respiratory distress due to mass effect or infection [5]. In some cases, CPAM may be misdiagnosed as neonatal pneumonia, particularly when symptoms persist despite appropriate antibiotic therapy [6]. Large lesions may also cause significant mediastinal shift, leading to the displacement of intrathoracic structures.

An important diagnostic challenge arises in differentiating true dextrocardia from cardiac dextroposition. Dextrocardia is a congenital condition characterized by a mirror-image positioning of the heart, often associated with situs abnormalities, whereas cardiac dextroposition refers to a secondary displacement of a structurally normal heart due to extracardiac causes such as lung lesions or mediastinal masses [7,8].

We report a case of CPAM revealed by neonatal respiratory distress and associated with cardiac dextroposition secondary to mediastinal shift, highlighting the importance of accurate interpretation of imaging findings to avoid diagnostic confusion.

## Case presentation

A 26-day-old male neonate, born at term to a 29-year-old mother (G4P4) from a non-consanguineous marriage, was admitted for cyanosis and neonatal respiratory distress. The pregnancy was reportedly well-followed, and delivery was vaginal. The early neonatal period was marked by respiratory distress initially attributed to a presumed pulmonary infection, for which the patient was hospitalized in another center and treated with broad-spectrum antibiotics (ceftriaxone, gentamicin, amikacin, followed by vancomycin), with transient clinical improvement. The patient required supplemental oxygen therapy but did not receive non-invasive or invasive mechanical ventilation. He was discharged five days prior to admission on continued antibiotic therapy, with oxygen saturation reported at 96% in room air. On the day of admission, during a follow-up transthoracic echocardiography, the infant developed worsening respiratory distress associated with generalized cyanosis, prompting referral to our institution.

**Figure 1:**
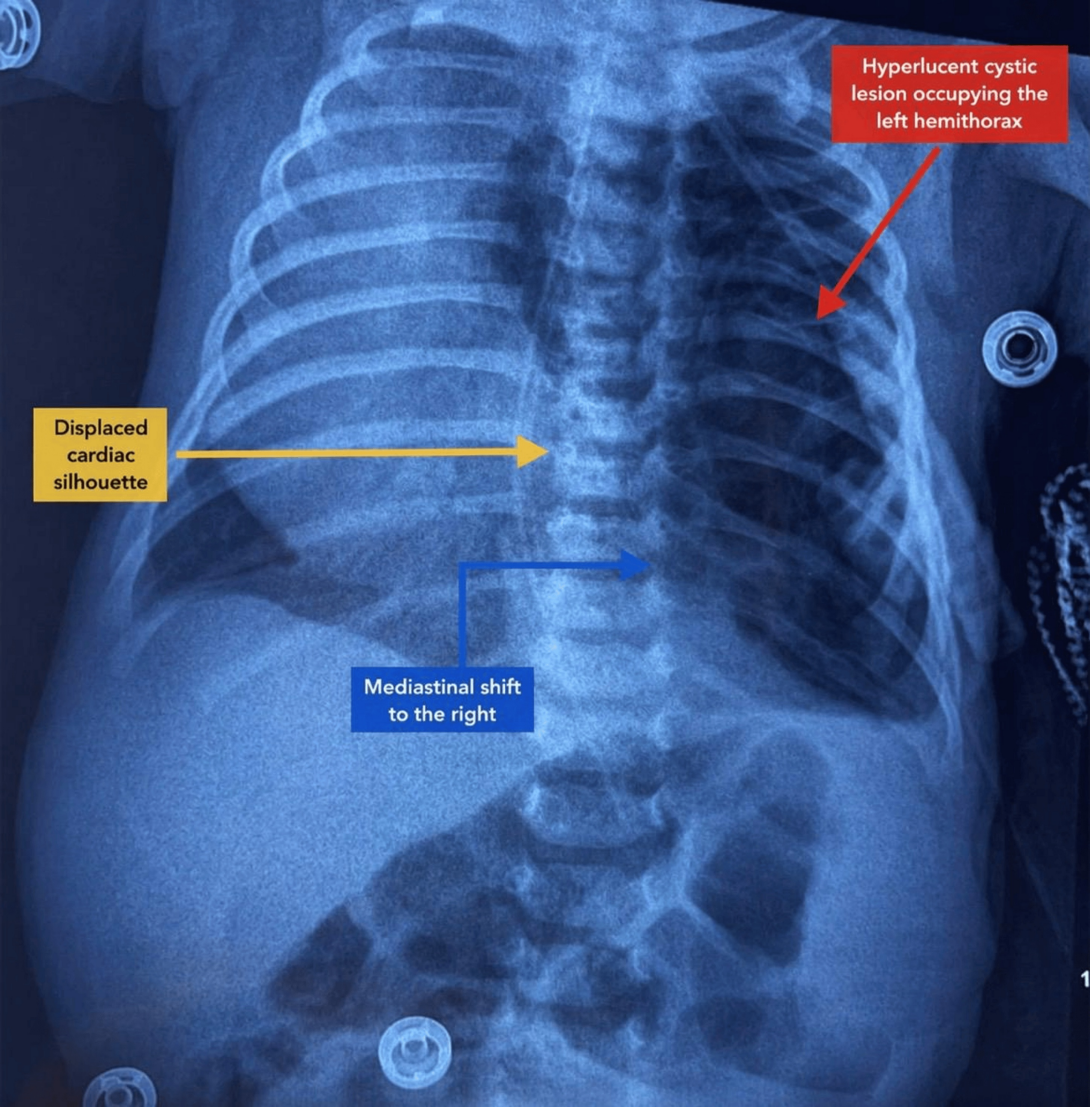
Anteroposterior chest radiograph of a 26-day-old neonate showing a large hyperlucent area occupying the left hemithorax (red arrow) There is a marked mediastinal shift toward the right (blue arrow) with displacement of the cardiac silhouette (yellow arrow), mimicking dextrocardia.

On admission, the neonate was conscious, reactive, and well-toned, with a weight of 3800 g (approximately 50th-75th percentile for age), length of 54 cm (approximately 75th percentile), and head circumference of 39 cm (above the 90th percentile). He presented with significant respiratory distress, characterized by tachypnea (68 breaths per minute), oxygen saturation of 65% in room air, and a Silverman score of 4/10, with thoraco-abdominal asynchrony and intercostal retractions, despite remaining conscious and reactive, likely reflecting a progressive onset of hypoxemia. Cardiovascular examination revealed tachycardia at 170 beats per minute, with heart sounds best heard over the right hemithorax, suggestive of dextrocardia, without murmur. Neurological examination was normal, with preserved primitive reflexes, including the sucking reflex, and a normotensive anterior fontanelle. Abdominal examination was unremarkable, with no hepatosplenomegaly, and the external genitalia were normal. No dysmorphic features or associated congenital malformations were identified. Chest radiography revealed a large hyperlucent cystic lesion occupying the left lung field, associated with significant mediastinal shift toward the right and an apparent right-sided cardiac silhouette (Figure 1).

**Figure 2:**
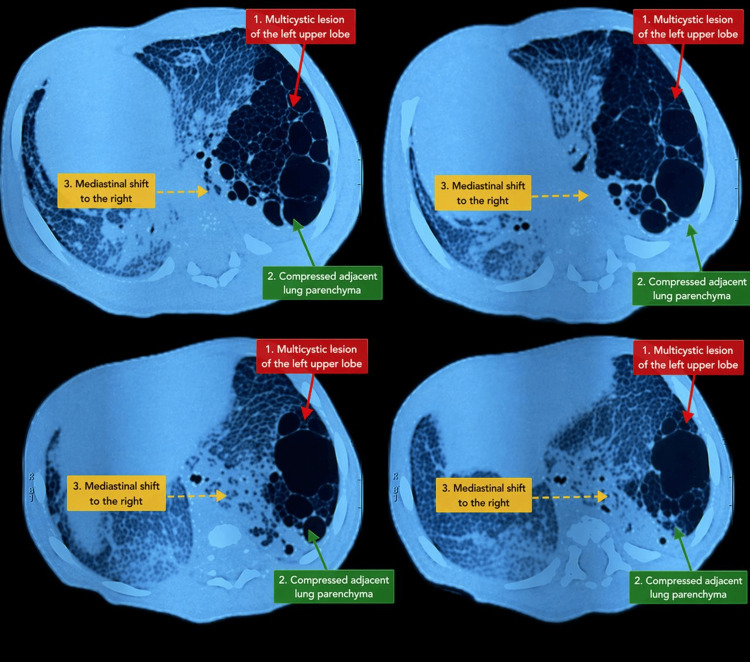
Axial chest CT images demonstrating a multicystic lesion of the left upper lobe (1), composed of multiple air-filled cysts of variable sizes, causing compression of the adjacent lung parenchyma (2) and a marked mediastinal shift to the right (3), consistent with congenital pulmonary airway malformation

Transthoracic echocardiography demonstrated situs solitus with a rightward cardiac position, normal atrioventricular and ventriculo-arterial concordance, and no structural congenital heart disease. A patent ductus arteriosus measuring 3 mm with a left-to-right shunt was noted, along with pulmonary hypertension (estimated pulmonary artery pressure of 65 mmHg) and biventricular hypertrophy with preserved systolic function. These findings support cardiac displacement rather than true dextrocardia. Thoracic computed tomography demonstrated a multicystic lesion involving the left upper lobe, composed of multiple air-filled cysts of variable sizes, causing compression of adjacent lung parenchyma and marked mediastinal shift with rightward displacement of the heart (Figure 2).

These findings were highly suggestive of CPAM, most consistent with Stocker type I. The rightward cardiac position was interpreted as cardiac dextroposition secondary to mediastinal shift, rather than true congenital dextrocardia. Despite supportive management, including oxygen therapy, the patient initially showed clinical improvement without the need for non-invasive or invasive mechanical ventilation. However, the clinical course was subsequently marked by progressive respiratory deterioration complicated by severe pulmonary infection, ultimately resulting in the patient’s death before surgical intervention could be performed.

## Discussion

Thoracic computed tomography plays a pivotal role in confirming the diagnosis of CPAM by providing detailed anatomical characterization of the lesion and its relationship with mediastinal structures [9]. In our case, CT imaging demonstrated a multicystic lesion of the left upper lobe with significant mediastinal shift, explaining the rightward displacement of the heart observed on clinical examination and chest radiography.

An important diagnostic consideration is the distinction between true dextrocardia and cardiac dextroposition. Dextrocardia refers to a congenital mirror-image positioning of the heart, whereas cardiac dextroposition corresponds to a secondary displacement of a structurally normal heart due to extracardiac causes such as lung lesions or mediastinal masses. In our patient, echocardiography confirmed situs solitus with normal cardiac anatomy, supporting a diagnosis of cardiac dextroposition secondary to mediastinal shift rather than true dextrocardia.

The lesion was most consistent with CPAM type I, the most frequent subtype, typically characterized by large cysts greater than 2 cm in diameter and a relatively favorable prognosis [4]. According to the Stocker classification, CPAM is divided into five types (0 to 4) based on airway origin and cyst size: type 0 (tracheobronchial), type I (large cysts), type II (smaller cysts), type III (microcystic or solid appearance), and type IV (large peripheral cysts). In our case, the presence of multiple large air-filled cysts on CT imaging supports the diagnosis of type I CPAM.

The differential diagnosis of cystic lung lesions in neonates includes pulmonary sequestration, congenital lobar emphysema, and bronchogenic cysts, all of which require careful radiological distinction due to differences in management and prognosis [10].

Management strategies for CPAM depend on clinical presentation. Surgical resection is generally recommended, even in asymptomatic patients, due to the risk of recurrent infections and potential malignant transformation, particularly into pleuropulmonary blastoma [11,12]. In symptomatic neonates, as in our case, early surgical intervention is often indicated. In our patient, initial clinical improvement under oxygen therapy without the need for mechanical ventilation likely reflected temporary control of a superimposed pulmonary infection. However, the clinical course was subsequently unfavorable, with progressive respiratory deterioration complicated by severe pulmonary infection, ultimately resulting in death before surgical management could be undertaken.

Congenital lesions, such as CPAM, may remain initially stable but can deteriorate in the presence of secondary infection or increasing pulmonary hypertension. The pulmonary hypertension observed in our patient may be explained by chronic hypoxia and compression of the pulmonary vasculature secondary to the mass effect of the lesion [5].

Overall, this case highlights the importance of considering CPAM in neonates with persistent respiratory distress and emphasizes the need to accurately distinguish cardiac dextroposition from true dextrocardia using multimodal imaging to avoid diagnostic confusion and guide appropriate management.

## Conclusions

This case highlights a rare presentation of congenital pulmonary airway malformation (CPAM) revealed by neonatal respiratory distress and associated with apparent dextrocardia secondary to mediastinal displacement. It underscores the importance of considering congenital pulmonary malformations in neonates with persistent or atypical respiratory symptoms, particularly when the response to antibiotic therapy is incomplete. Multimodal imaging, including chest radiography, echocardiography, and computed tomography, plays a crucial role in establishing the diagnosis and distinguishing true cardiac malposition from secondary displacement. Early recognition of this condition is essential to guide appropriate management and prevent potential complications.
